# Data concerning the rheological behavior of high methoxyl pectin during gelation process

**DOI:** 10.1016/j.dib.2018.04.064

**Published:** 2018-04-24

**Authors:** Daniela Giacomazza, Donatella Bulone, Pier Luigi San Biagio, Rosamaria Marino, Romano Lapasin

**Affiliations:** a CNR – Istituto di BioFisica (IBF), Via U. La Malfa153, I-90146 Palermo, Italy; b Silvateam Food Ingredients, Via M. Polo, 72/74, I-87036 Rende, CS, Italy; c Dipartimento di Ingegneria e Architettura, Università degli Studi di Trieste, Piazzale Europa, I-34127 Trieste, Italy

## Abstract

The present data concern the structuring kinetics of aqueous high methoxyl pectin (HMP) solutions at acid pH (3.1), constant pectin concentration (0.2% w/w) and sucrose concentrations ranging from 56 to 65% w/w. Consecutive frequency sweep was applied to samples immediately after their preparation. The generalized Maxwell (gM) model was used to describe the change of the mechanical spectra for each different sucrose concentration and to determine the viscoelastic parameters controlling the gelation of the HMP solutions. The viscosities in the sol region are explored in the range 0 to 55% 0 to 40% (w/w) sucrose concentration.

**Specifications Table**

**Table:** 

Subject area	*Physics; Chemical Engineering*
More specific subject area	*Polymer behavior; Hydrogels*
Type of data	*Rheological mechanical spectra; Rheological models*
How data was acquired	*TA Instruments AR-1000 Rheometer*
Data format	Experimental and analyzed data
Experimental factors	The samples were prepared at different sucrose concentrations and placed in the measuring device immediately after their preparation.
Experimental features	The gelation process of the High Methoxyl Pectin has been analyzed in terms of generalized Maxwell Model.
Data source location	*Palermo, Italy*
	*Trieste (Italy)*
Data accessibility	*Data are provided in the present article.*

**Value of the data**

• Gelation mechanism of High Methoxyl Pectin solutions.
• Time evolution of the viscoelastic parameters.
• Role of sucrose in the 3D network of high methoxyl pectin.

## 1 Data

**Fig. 1:**
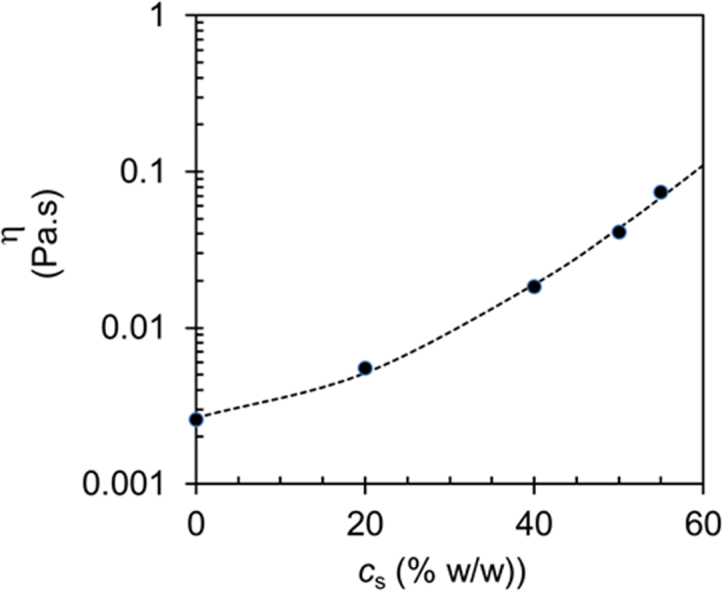
Viscosity vs sucrose concentration. Viscosity values at the temperature of 20 °C for samples prepared with the same pectin concentration (0.2% w/w) and different sucrose concentrations (0, 20, 40, 50, 55% w/w).

The present data concern rheological experiments and analysis performed on pectin aqueous solution (0.2% w/w) in presence of increasing sucrose concentrations (from 0% up to 65% w/w) in the sol (Fig. 1) and self-assembled regions.

**Fig. 2:**
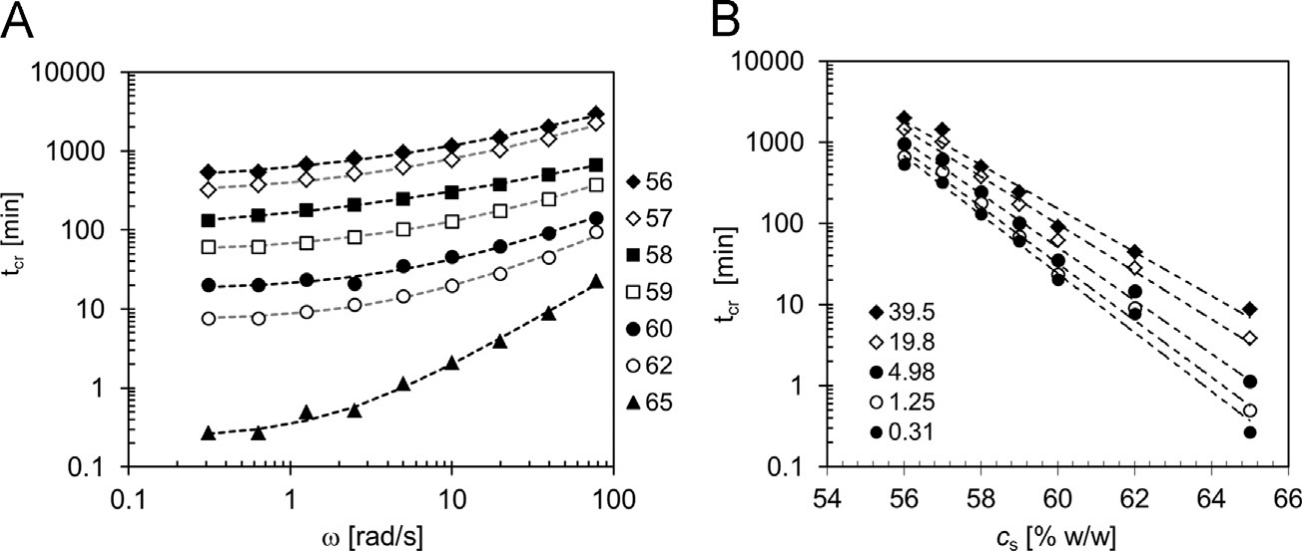
Crossover times. A) Frequency dependence of crossover time at different sucrose concentrations (from 56 to 65% w/w). The dotted lines are obtained from best fit equation $t_{\mathrm{cr}}$=$t_{\mathrm{cr},0}+k\omega^{q}$. B) Dependence of crossover time on sucrose concentration at different frequencies (from 0.31 to 39.5 rad/s). The straight lines show the exponential trend.

In Fig. 2A the crossover times for the different sucrose concentrations are reported as a function of the frequency. In Fig. 2B, the behavior of the crossover times at different frequency values is shown as a function of the sucrose content of the samples. In both figures, *t*_cr_ values lower than 10 min are obtained by extrapolation of the kinetic data with Eq. (1).

(1) $$G\;\left(t\right)=G_{0}+\frac{\left(G_{\infty }\;-\;G_{0}\right)\cdot {\left(t/t_{c}\right)}^{n}}{1+\;{\left(t/t_{c}\right)}^{n}}$$

**Fig. 3:**
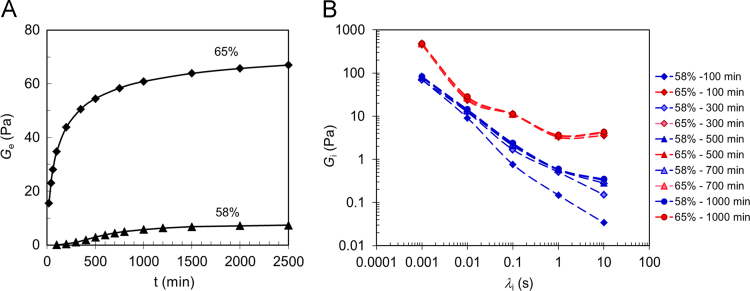
Generalized Maxwell (gM) model analysis. Time evolution of the gM parameters for systems 58% and 65%. A) Equilibrium modulus (*G*_*e*_) vs process time; B) time relaxation spectra at different process times.

**Fig. 4:**
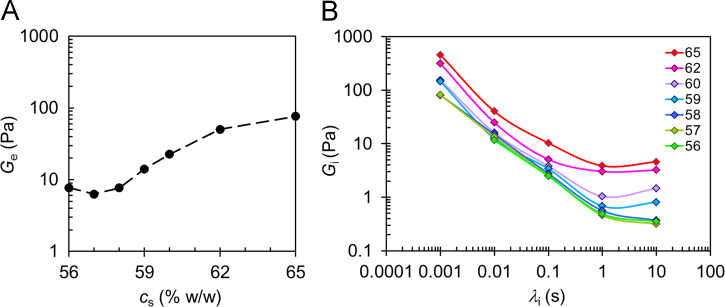
gM asymptotic behavior. Dependence of the asymptotic gel responses on sucrose concentration as described by gM model parameters. A) Equilibrium modulus (*G*_*e*_) and B) time relaxation spectrum *G*_i_ (*λ*_i_*)*.

The generalized Maxwell (gM) model has been used to describe the change of the mechanical spectra for each different sucrose concentration and to determine the viscoelastic parameters controlling the gelation of the HMP (Fig. 3A and B; Fig. 4A and B). They are the equilibrium modulus, G_e_, representing the asymptotic value of the storage modulus for ω → 0, *G*_*i*_ and *λ*_*i*_ values corresponding to the relaxation modulus and the relaxation time of the *i*^th^ Maxwell element, respectively. Data analysis is in perfect agreement with that performed with Friedrich-Braun model [1]. and highlights the role of sucrose in the structuring process of the High Methoxyl Pectin 3D network [2]..

## 2 Experimental design, materials and methods

### 2.1 Viscosity measurements

The viscosity measurements were performed at 20 °C with a rotational controlled stress rheometer (AR-1000, TA Instruments, UK) by using a titanium cone-plate geometry (angle 0.0174 rad, radius 20 mm, truncation 26 μm). Shear rate ramps were performed in the sol region for samples prepared at the sucrose concentrations of 0, 20, 40, 50, and 55% w/w in the range 50–6500 s^-1^.

### 2.2 Rheological measurements

Samples at different sugar concentrations (56, 57, 58, 59, 60, 62, and 65% w/w) were prepared by heating at 100 °C for 10 min under stirring the potassium citrate buffer (12 mM, 22.5% w/w, pH 3.4) with sucrose and the right water quantity. A constant amount of the pectin stock solution was added to obtain a final concentration of 0.2 w/w and boiled for further 10 min. Finally, a few microliters of 50% citric acid solution was added to adjust the pH value to 3.1. The rheological monitoring of the gelation process was performed at 20 °C with a rotational controlled stress rheometer (AR-1000, TA Instruments, UK), equipped with a standard-size double concentric aluminum cylinder (rotor outer radius 21.96 mm, rotor inner radius 20.38 mm, stator outer radius 20.00 mm, cylinder immersed height 59.50 mm, gap 500 μm). A circulating bath thermostat was used for temperature control.

A series of consecutive frequency sweeps (logarithmic sequence of increasing values from 0.02 up to 30 Hz, time needed per sweep: 15 min) was carried out at constant strain amplitude (4 × 10^-3^) for very prolonged times, ranging from 1400 (65% system) to 8500 min (56% system). The air/sample interface was coated with silicon oil to avoid sample evaporation.
