# Supplementary material for: Data concerning the rheological behavior of high methoxyl pectin during gelation process
Source: Data Brief. 2018 Apr 24;18:1628–31. doi: 10.1016/j.dib.2018.04.064 (PMC5998181; doi:10.1016/j.dib.2018.04.064)
Supplement: Supplementary file 1 — Supplementary material [file mmc1.rtf]

Conflict of Interest
The Authors declare no conflict of interest.
